# Understanding the unexplained: The magnitude and correlates of individual differences in residual variance

**DOI:** 10.1002/ece3.7603

**Published:** 2021-05-03

**Authors:** David J. Mitchell, Christa Beckmann, Peter A. Biro

**Affiliations:** ^1^ Centre for Integrative Ecology School of Life & Environmental Sciences Deakin University Geelong VIC Australia; ^2^ Department of Zoology/Ethology Stockholm University Stockholm Sweden; ^3^ School of Science and Health Western Sydney University Parramatta NSW Australia; ^4^ Hawkesbury Institute for the Environment Western Sydney University Penrith NSW Australia

**Keywords:** animal personality, behavioral plasticity, behavioral predictability, behavioral reaction norm, intraindividual variability

## Abstract

Behavioral and physiological ecologists have long been interested in explaining the causes and consequences of trait variation, with a focus on individual differences in mean values. However, the majority of phenotypic variation typically occurs within individuals, rather than among individuals (as indicated by average repeatability being less than 0.5). Recent studies have further shown that individuals can also differ in the magnitude of variation that is unexplained by individual variation or environmental factors (i.e., residual variation). The significance of residual variation, or why individuals differ, is largely unexplained, but is important from evolutionary, methodological, and statistical perspectives. Here, we broadly reviewed literature on individual variation in behavior and physiology, and located 39 datasets with sufficient repeated measures to evaluate individual differences in residual variance. We then analyzed these datasets using methods that permit direct comparisons of parameters across studies. This revealed substantial and widespread individual differences in residual variance. The magnitude of individual variation appeared larger in behavioral traits than in physiological traits, and heterogeneity was greater in more controlled situations. We discuss potential ecological and evolutionary implications of individual differences in residual variance and suggest productive future research directions.

## Graphical abstract

Animals are typically quite unpredictable in behavior with the majority of variance unexplained. We returned to raw data of previously published work, and found almost ubiquitously that individuals vary in their predictability. We discuss potential ecological and evolutionary implications of individual differences in residual variance and suggest productive future research directions.

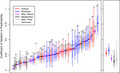

## 1 INTRODUCTION

Evolutionary ecologists have long been interested in the magnitude of trait variation, and the causes and maintenance of such variation. In contrast to morphological and life‐history variation, behavioral and physiological traits offer additional challenges due to their lability over short temporal scales. While individuals commonly differ in their mean level trait, as evidenced by significant repeatability (Bell et al., 2009; Fanson & Biro, 2018; Schoenemann & Bonier, 2018; White et al., 2013), these traits can change quickly in response to the current environmental context (known as “contextual plasticity”; Biro et al., 2010; Careau, Gifford et al., 2014; Fürtbauer et al., 2015; Westneat et al., 2011) or temporally (Biro et al., 2014; Carter et al., 2012; Martin & Réale, 2008). Even after accounting for these sources of systematic variation, considerable variation often remains. Contributing factors to this residual variation are many, including those with biological significance such as unaccounted for variation in environmental conditions, internal state factors and instabilities in biological processes, or simple measurement error owing to imprecision in our methods.

This residual intraindividual variability (rIIV) can differ between individuals, so that some individuals are predictable about their mean, relative to others which at the same time, situation and context are unpredictable (Briffa et al., 2013; Stamps et al., 2012; Westneat et al., 2013). While a long‐known phenomenon in psychology (MacDonald et al., 2006; Ram et al., 2005), individual variation in rIIV has a shorter history in evolutionary ecology (Stamps et al., 2012). Studies in evolutionary ecology to date suggest that rIIV shows distinct among‐individual variation (Briffa, 2013; Mitchell et al., 2016; Montiglio et al., 2015; Stamps et al., 2012; Westneat et al., 2013). By obtaining multiple clusters of observations separated through time, studies have found these individual differences in rIIV to be consistently expressed through time (Biro & Adriaenssens, 2013; Highcock & Carter, 2014). Further, studies in plants have indicated epigenetic effects on the amount of rIIV exhibited (Herrera, 2017), while studies in animals have found additive genetic components (i.e., heritability; Martin et al., 2017; Prentice et al., 2020; Rönnegård et al., 2013). Together, these observations indicate rIIV should be viewed as part of a more complex trait structure, representing a distribution of scores an individual may express in a given context and point of time (Fleeson, 2001). Perhaps for this reason, interest in residual variance is growing across diverse disciplines of biology, including the fields of animal ecology, plant ecology, and animal breeding (reviewed respectively in Herrera, 2017; Mulder et al., 2008; Westneat et al., 2015).

Many ideas have been put forward to suggest processes which may affect mean rIIV. For instance, researchers have speculated that rIIV could affect predictability in biotic interactions; being variable may reduce predictability to a predator and therefore reduce the risk of capture (Briffa, 2013; Domenici et al., 2008; Jones et al., 2011), while increased predictability may aid in cooperative and social interactions where animals aim to coordinate behaviors (Wolf & Krause, 2014). For social species, where safety is conferred through social behaviors—for example, through confusion effects (Landeau & Terborgh, 1986), or shared information (Ward et al., 2011)—these two opposing forces may give rise to multiple strategies and therefore among‐individual variation in rIIV. Models of behavior based on game theory, such as the “prisoner's dilemma” (Axelrod & Hamilton, 1981) and “hawks and doves” (Smith & Price, 1973), are based on repeated interactions of social partners within a population. These models have an implicit assumption of predictability in individual behavior. For instance, during predator inspection an individual may use a social partner's past behavior to predict the current behavior (Milinski et al., 1990). The existence of unpredictable individuals would falsify this assumption and could therefore fundamentally change the dynamics and possible strategies of such games (Wolf et al., 2008).

Residual variance could also arise through behavioral plasticity. Papers have previously suggested that rIIV may be created by plastic responses to unobserved or uncontrolled environmental stimuli (Stamps et al., 2012; Westneat et al., 2015) and variation in rIIV would result if individuals varied in response to these stimuli, or if individuals differed in the amount of environmental variation they are exposed to. Under such assumptions, one would predict greater individual variation in rIIV in studies that have less control over conditions (Goold & Newberry, 2017; Westneat et al., 2011), such as field studies. Unaccounted for effects could also include internal stimuli, such as hormone levels or reproductive state (Kim et al., 2018), and thus, individual differences in rIIV may arise through endogenous plasticity (Stamps, 2016). Such endogenous effects would be expected to affect laboratory and field studies equally.

Despite the potential importance of this residual variance, few studies consider individual variation in rIIV, or have the requisite sampling regimes to robustly quantify it. Even among studies explicitly focused on rIIV, there is often little comparability, due to the use of different statistical techniques employed. Initially, studies used a two‐step approach, whereby a mean model was fit and the residuals were extracted for use in a second analysis (see Biro & Adriaenssens, 2013; Stamps et al., 2012). Results from this method are sensitive to small or uneven sample sizes, and only comparable when fit to standardized data (Cleasby et al., 2015). A second option is to fit individual‐specific residual variances (see Briffa et al., 2013; Highcock & Carter, 2014; Montiglio et al., 2015), though this technique yields no effect size estimate on the variance in rIIV and is very parameter heavy, fitting one residual variance parameter per individual.

Recently, a “best‐practice” analysis has been introduced to ecologists (Cleasby et al., 2015; Westneat et al., 2013), though it has a longer history of use in other fields (Felleki et al., 2012; Mulder et al., 2008; Rönnegård et al., 2013; Smyth, 1989). The double‐hierarchical generalized linear model (DHGLM) estimates the among‐individual variance in rIIV, using a more parsimonious modeling technique, with greater power and comparability among studies (Cleasby et al., 2015). Initially, these analyses required coding into the Bayesian programs “JAGS” (Plummer, 2004) or “Stan” (Stan Development Team, 2016), which provided a large barrier to entry as they required considerable programming (e.g., Mitchell et al., 2016; Westneat et al., 2013). The recent development of the R package “brms” (Bürkner, 2017) as an interface to “Stan” has made coding these models much more user‐friendly (e.g., Hertel, Niemelä, et al., 2020; Mitchell, Beckmann, et al., 2020). However, results from DHGLMs are not comparable with older methods—leading to a literature which is currently disjunct, with patterns across datasets hard to distinguish. There is little consensus on the commonality or magnitude of individual variation in rIIV, or how rIIV relates to other aspects of the phenotype—be it the mean level trait, or rIIV in other traits. The topic is therefore currently in need of being brought into a more unified framework.

To address these issues, we reviewed the literature for studies of rIIV. To more broadly address the commonality and magnitude of these effects, we also gathered well‐sampled datasets that did not consider individual differences in rIIV. We used the raw data from papers published on behavioral and physiological traits to quantify (a) the magnitude of individual differences in rIIV, (b) whether rIIV commonly correlates with the individual's trait mean; and where possible (c) whether rIIV correlates among individuals across different traits. In light of these analyses, we then discuss the biological factors, which may lead to variation in rIIV, and covariance of individual rIIV with individual “personality” (means).

## 2 AVAILABLE DATA ON RESIDUAL VARIANCE

Our initial aim was to review the literature on individual variation in rIIV. A secondary aim to meta‐analyze these results grew organically out of this endeavor. We collated suitable datasets from the literature looking broadly for animal ecology papers quantifying individual variation in behavior or physiology (typically metabolism or hormone traits). We aimed for well‐sampled datasets, with a minimum of 20 individuals, sampled at least five times each, which was viewed as a minimum to yield some power to test for variation in rIIV (Cleasby et al., 2015). Data with frequent artificial ceiling or floor values (e.g., maximum latencies to emerge) were not considered for inclusion. Due to these factors, our literature search was largely haphazard and in addition occurred over a period of the last 5 years where we created Google Scholar alerts set to papers citing (Stamps et al., 2012) and our own papers. An attempt to retrofit a formal literature search demonstrated that the definition of logical search terms was difficult, as we were largely interested in finding datasets based on sampling regimes—not a specific research topic. For instance, searching “behavioural predictability,” “intraindividual variability,” and “intraindividual variation” in Web of Science and filtering to related biological fields returned just 173 unique papers, with 12 usable datasets—all of which had already been located with our informal search. Due to the informal search, we caution that results from the meta‐analytical models should be taken as only indicative, with likely unknown biases arising due to the datasets we located. In total, we collated data from 39 studies, totaling 64 estimates of CV_P_.

The compiled datasets were highly heterogeneous in the traits and taxa they represented including eight different mammal species, six fish species, three crustacean species, three bird species, two reptile species, one gastropod species, one amphibian species, and one cnidarian species. Guppies (*Poecilia reticulata*) were the best represented species, with seven datasets (16 estimates) from four independent laboratories, followed by hermit crabs (*Pagurus bernhardus*; six studies and nine estimates all from Mark Briffa's laboratory). Many estimates represented either activity (20 estimates) or boldness (20), with additionally three studies and seven estimates of metabolic traits, two studies and estimates of hormone traits, among a number of other traits.

Datasets were (re‐)analyzed with “brms” (Bürkner, 2017) where required, and R code and output can be found on the open science framework (OSF; Mitchell, 2021). Each dataset was standardized to a mean of 0 and standard deviation of 1 before analysis. This transformation does not affect the parameters of interest, but aided in fitting standardized uninformative priors. We aimed to calculate the variation in rIIV once obvious systematic variation was accounted for using fixed and random effects; this is an important point as we follow Stamps et al. (2012) in the view that individual rIIV should be studied only after obvious fixed and random effects are accounted for in the models. Therefore, contextual or temporal plasticity in the mean was included (i.e., random slope effects for time and/or context) where applicable. From each analysis, we exported the CV_P_ (Equation 1) for meta‐analysis. This estimate is reported conditionally, after heterogeneity was accounted for that was readily explained by fixed effects in the residual model. Some studies model the residual variance rather than residual standard deviation (e.g., Martin et al., 2017; Prentice et al., 2020) and required conversion. On the log‐scale, there is a linear relationship between variance and standard deviations, and the variance in the rIIV can therefore be easily converted as $\omega_{\sigma }^{2}=\frac{\omega_{\sigma^{2}}^{2}}{4}$ (O'Dea et al., 2020).

(1) $${\text{CV}}_{P}=\sqrt{\exp\left(\omega_{\sigma }^{2}\right)‐1}$$

We also extracted the correlation of rIIV (a random intercept in the residual model) with the mean level trait (at mean‐centered random slopes where they were present). In a few instances, we located studies that measured more than one trait, which permitted us to examine whether animals are generally (un)predictable across multiple traits. In those cases, we ran multivariate DHGLMs as discussed in Ref. (Hertel, Royauté, et al., 2020; Mitchell, Beckmann, et al., 2020) and additionally extracted the among‐individual correlation in rIIV across traits, and among‐individual correlation in means of the same traits (details below in the section “Correlation of rIIV across traits”).

Due to the heterogeneity in the taxa and traits considered (while not having large samples), we ran only simple meta‐analyses. Single estimates of CV_P_ presented a modest positive skew of their credible distribution, though transformation (either log or square root) created a worse negative skew. As the positive skew was minor and unlikely to cause a meaningful bias, we left the estimates untransformed. Among studies, however, there was a clear log‐normal distribution. To account for this, we modeled CV_P_ on the log‐scale through the use of the nonlinear commands in “brms” (Bürkner, 2017), which also constrained estimates and their uncertainty to positive values (see Mitchell, 2021, for analysis code). To understand general patterns in CV_P_, we ran four meta‐analytical models of increasing complexity, which aimed to (a) understand the grand weighted‐mean CV_P_ across all studies, (b) assess whether CV_P_ generally differs between behavioral (*n* = 53 estimates) or physiological traits (*n* = 11 estimates), (c) evaluate whether traits differ in their CV_P_ by fitting a fixed effect with five levels (boldness, activity, miscellaneous behavior, metabolism, and miscellaneous physiology), and (d) evaluate whether field studies display higher CV_P_ owing to the lack of control of environmental conditions (same as c, with additional fixed effect of field or laboratory). All models were run with a random intercept effect of estimate (to acknowledge variation in CV_P_ within studies), and variance was separated by trait type (i.e., behavioral or physiological) for models b‐d. Study identity was also fit as a random intercept as either multiple traits or subpopulations were sometimes present within a study. Similar analyses were conducted on the other extracted parameters, which we discuss below.

## 3 RESULTS AND DISCUSSION

### 3.1 Meta‐analysis of CV_P_

Analyses revealed that variation in rIIV was very common, with an overall predicted CV_P_ of 0.26 [95% Credible Intervals: 0.21, 0.31]. We did not pick up clear signs of publication bias in the studies considered, with no effect observed of whether the original publication considered variation in rIIV (see Mitchell, 2021, for full output). However, care should still be taken in interpreting these results due to the largely informal search. Behavioral traits had an overall CV_P_ of 0.27 [0.22, 0.33], while for physiological traits it was 0.17 [0.074, 0.29], though this difference was not significant (difference = −0.11 [−0.22, 0.022]). The lack of a difference was largely driven by a single hemolymph density study in shore crabs (*Carcinus maenas*; Fürtbauer, 2015) that showed unusually pronounced among‐individual variance in both means and variance, which inflated the variance in physiological studies and thus the imprecision. Removal of this study from the analysis demonstrated the large effect it had (difference: −0.13 [−0.22, −0.027]).

Contrary to the prediction that field studies would show greater variance in rIIV due to reduced control over conditions (Stamps et al., 2012; Westneat et al., 2015), field studies typically appeared to have lower estimates than laboratory studies (difference on log‐scale = −0.57 [−0.96, −0.17]). A lack of control over environmental conditions appeared to saturate intrinsic differences in rIIV, leading to lower estimates of CV_P_. If there is a strong population effect of an unmeasured environmental variable, with little individual variation in plasticity, this would add uniformly to the rIIV. As the CV_P_ is standardized by the mean rIIV, this would reduce the estimate of individual variation in rIIV, rather than add to it. However, care should be taken with this result as field studies typically used different assays; for example, studies of boldness were typically based on flight initiation distance (Allan et al., 2020; Highcock & Carter, 2014) rather than latency to emerge (e.g., Horváth et al., 2019; Stamps et al., 2012), and measures of activity were based on tracking over large spatial scales (e.g., Hertel, Niemelä, et al., 2020; Hertel, Royauté, et al., 2020) rather than tracking within an arena (e.g., Mitchell & Biro, 2017; Prentice et al., 2020).

**FIGURE 1:**
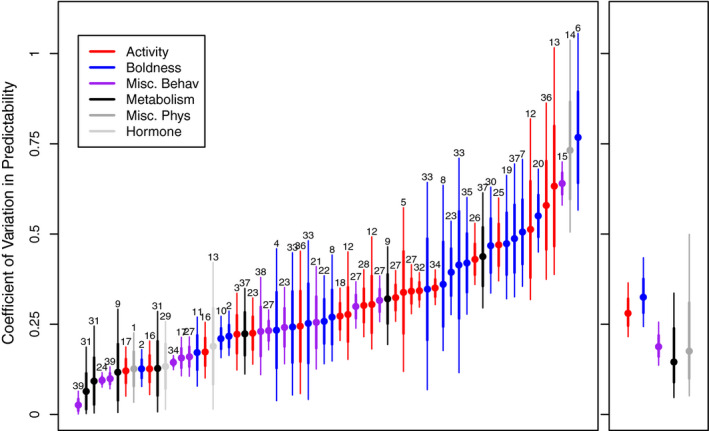
Displayed are the individual best estimates with one standard deviation of the credible distribution in bold and the 95% credible distributions shown by the thin lines. Different traits are assorted by color, and meta‐analytical models of model 3 are presented in panel b. Numbers above each estimate correspond to the study identity. 1: Western fence lizard (*Sceloporus occidentalis*; Adolph & Pickering, 2008), 2: chacma baboon (*Papio ursinus*; Allan et al., 2020), 3: mosquitofish (*Gambusia holbrooki*; Biro & Adriaenssens, 2013), 4: Ward's damselfish (*Pomacentrus bankanensis*; Biro et al., 2010), 5: guppy (*Poecilia reticulata*; Biro et al., 2016), 6: hermit crab (*Pagurus bernhardus*; Bridger et al., 2015), 7: hermit crab (*P. bernhardus*; Briffa, 2013), 8: hermit crab (*P*. *bernhardus*; Briffa et al., 2013), 9: house sparrow (*Passer domesticus*; Careau, Hoye, et al., 2014), 10: marsh periwinkle (*Littoraria irrorata*; Cornwell et al., 2018), 11: marsh periwinkle (*L. irrorata*; Cornwell et al., 2020), 12: house mouse (*Mus musculus*; Eisenmann et al., 2009), 13: three‐spined stickleback (*Gasterosteus aculeatus*; Fürtbauer et al., 2015), 14: shore crab (*Carcinus maenas*; Fürtbauer, 2015), 15: dog (*Canis familiaris*; Goold & Newberry, 2017), 16: guppy (*P. reticulata*; Herczeg et al., 2019), 17: brown bear (*Ursus arctos*; Hertel, Royauté, et al., 2020), 18: African elephant (*Loxodonta africanus*; Hertel, Niemelä, et al., 2020), 19: Namibian rock agama (*Agama planiceps*; Highcock & Carter, 2014), 20: slater (*Armadillidium vulgare*; Horváth et al., 2019), 21: fallow deer (*Dama dama*; Jennings et al., 2013), 22: three‐spined stickleback (*G. aculeatus*; Jolles et al., 2019), 23: guppy (*P. reticulata*; Kurvers et al., 2018), 24: yellow‐bellied marmot (*Marmota flaviventris*; Martin et al., 2017), 25: guppy (*P. reticulata*; Mitchell et al., 2016), 26: zebra fish (*Danio rerio*; Mitchell, Dujon, et al., 2020), 27: guppy (*P. reticulata*; Mitchell, Beckmann, et al., 2020), 28: guppy (*P. reticulata*; Mitchell, Lefèvre, et al., 2020), 29: eastern chipmunk (*Tamias striatus*; Montiglio et al., 2015), 30: hermit crab (*P. bernhardus*; Nanninga et al., 2020), 31: (Norin & Gamperl, 2017), 32: zebra fish (*D. rerio*; O'Dea et al., 2020), 33: (Osborn & Briffa, 2017), 34: guppy (*P. reticulata*; Prentice et al., 2020), 35: hermin crab (*P. bernhardus*; Stamps et al., 2012), 36: agile frog tadpole (*Rana dalmatina*; Urszán Tamás et al., 2018), 37: hermit crab (*P. bernhardus*; Velasque & Briffa, 2016), 38: red‐winged blackbird (*Agelaius phoeniceus*; Westneat et al., 2013), 39: pied flycatcher (*Ficedula hypoleuca*; Westneat et al., 2017

While variation in rIIV was nearly ubiquitous, these were highly variable across studies and estimates, ranging from essentially 0 (0.03) to highly pronounced and visually obvious 0.76 (Figure 1a). This was evident in the meta‐analytical models, with large variance among studies, and among different traits or subpopulations within studies. This variation is pronounced, with many studies showing CV_P_ estimates below 0.25, which can be difficult to distinguish from the null (compare simulations in Figure 2a,b). However, among‐individual variation in rIIV could also be very large, as indicated by CV_P_ estimates of 0.5 to 0.75 (Figure 2c,d, respectively).

**IGURE 2:**
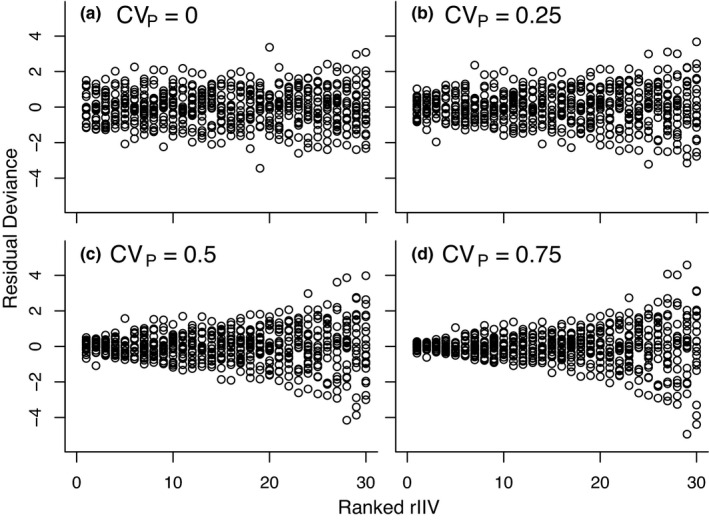
FShown are simulated data to illustrate what individual variation in rIIV looks like, under different effect sizes of CV_P_. Thirty Individuals are simulated, each with 20 observations. Individuals are then ranked from smallest to largest variance, and simulated data points are shown as a residual deviance from 0. When sample sizes are reduced, the heterogeneity appears greater, especially at the null (a). Code to replicate these simulations can be found in the Appendix S1

Different factors of the endogenous state could create variability over different periods of time (Mitchell, Dujon, et al., 2020). For instance, neural activity can change very quickly and cause variability over short time periods (Ribeiro et al., 2016), while energy availability may change over hours or days—thus creating variability over a longer period of time (Mitchell & Biro, 2017). The estrous cycle creates variability over the course of three to 4 days in mice (Carmichael et al., 1981), which regulates activity levels (Gerall et al., 1973), creating rIIV from endogenous effects in both hormones and behavior. Studying individual‐level cycles may therefore help us understand the proximate causes of rIIV, as differences in amplitude, frequency, and sensitivity to these internal cycles may create individual differences in behavioral and physiological rIIV. More broadly, studying these temporal patterns may illuminate the most important contributing proximate causes to the maintenance of consistent individual variation in these labile traits.

### 3.2 Meta‐analysis of correlation of rIIV with the mean trait value

Many ideas have been posited as to why we may expect the mean trait value to covary with rIIV (Highcock & Carter, 2014; Mitchell et al., 2016; Westneat et al., 2015), which we discuss below. We focused our analyses on boldness (*n* = 19 estimates) and activity rates (*n* = 20 estimates), where we had a reasonable amount of data to assess generality in these correlations. Most of the boldness assays related to latency to emerge following a fright stimulus. Time perception is typically thought to scale logarithmically (Staddon & Higa, 1999), while log‐scaling is also a reasonable assumption in flight initiation distance due to being bounded by 0, as well as likely perception error arising due to larger distances being harder for the animal to estimate accurately. As such, all boldness behaviors were analyzed on the log‐scale (except Jolles et al., 2019, which was proportion in center of an arena). Activity rate analyses were more heterogeneous, with some datasets representing proportion of time moving (Biro & Adriaenssens, 2013; Herczeg et al., 2019), while others were based on distance moved (e.g., Hertel, Royauté, et al., 2020; Mitchell & Biro, 2017) and were transformed where appropriate to achieve symmetry and meet distributional assumptions. Transformations in the study of rIIV are highly important as they set the null prediction of the covariance in the mean, affecting the correlation of “personality” with rIIV, and also the magnitude of variance in rIIV (i.e., CV_P_; Yang et al., 2011). Correlation coefficients extracted from these analyses were transformed to Fisher's *Z*‐correlation (Equation 2) to normalize the data for meta‐analysis.

(2) $$Z=\frac{1}{2}\ln\left(\frac{r+1}{r‐1}\right)$$

There was a slight tendency for bolder individuals to exhibit higher rIIV, though this effect was nonsignificant overall (*r* = 0.24 [−0.007, 0.47]). For activity rates, there was little indication for a general correlation of more active individuals being more consistent (lower rIIV; *r* = −0.15 [−0.34, 0.046]). For both traits, there was little consistency among estimates from differing studies—indicating little generality among taxa, populations, or metrics of these behaviors. This was indicated by the substantial variation among estimate (boldness: *σ*_Fisher's *Z*_ = 0.47 [0.28, 0.75], activity: *σ*_Fisher's *Z*_ = 0.38 [0.23, 0.6]). Both trait types showed no correlation between the mean and rIIV (*r* = 0) to be within one standard deviation of the mean coefficient, indicating there was variance not just in the magnitude of the correlation, but also the direction.

A previous study that observed a positive correlation of boldness and rIIV speculated this may counterbalance risk associated with the mean phenotype (Highcock & Carter, 2014). However, such functional unpredictability would be limited to systems where interactions are likely to be repeated through time at the individual level (e.g., Wittemyer et al., 2008). Where repeated interactions are rare or absent, predators may be more likely to make predictions based on population‐level means and variances, leading to apostatic selection (Clarke, 1969). This would not predict a mean–variance relationship among individuals. There are additionally numerous counter‐prevailing forces (discussed below), which are likely to drive correlations between the mean and variance, leading to the observed variation among systems and trait metrics considered.

When selection gradients are nonlinear, we need to understand both the mean and variance of the trait score. This line of reasoning has led researchers to speculate that extreme values of behavior could often be more consequential than the mean (Adolph & Pickering, 2008; Lichtenstein et al., 2017). This is conveniently demonstrated in a predator–prey scenario, where the occasional “mistake” may ultimately dictate survival and fitness of prey. The importance of the shape of the relationship to selection on trait variance is effectively described by “Jensen's inequality” (Ruel & Ayres, 1999). Jensen's inequality simply states that when *x* (the trait) and *y* (the fitness related consequence) have a linear relationship, the mean of *y* can be directly calculated from the mean of *x*—without regard for the variance. When the relationship is nonlinear, the mean of *y* then becomes a function of both the mean and variance of *x*.

In antipredator behaviors such as flight initiation distance or latency to emerge, the relationship between the expressed behavior at each instance and consequence follows a sigmoidal function as the consequence is binomial (the individual was either captured or not). For bold individuals (those that exhibit a short latency or flight initiation distance), high variability would be more likely to lead to instances of highly costly behavior, and death. For low‐risk individuals, even extreme deviations from their mean behavior are unlikely to lead to a costly outcome. While in the example of boldness to predator survival, a meta‐analysis did not reveal a correlation between boldness and survival at the among‐individual level (Moiron et al., 2020), this does not speak to the relationship at the within‐individual level. Indeed, this variance sensitivity could contribute to disentangling the relationship between individual mean behavior and fitness. If the outcome is mediated by other traits (e.g., escape performance), this too could drive correlations between the other traits and rIIV in the boldness behavior. The effect would be for selection to drive a correlation of bolder individuals being more consistent. Where the relationship between the behavior and the consequence instead follows a linear relationship, the overall mean “consequence” is dependent only on the mean behavioral expression. Such linear relationships may arise between foraging activity and acquired resources, or metabolic traits and energy expenditure, and would be predicted not to generate a relationship between the mean and rIIV.

Variation in rIIV is also likely to arise from nonfunctional processes. Animals may be constrained by proximate physiological traits to the range of behavioral expression they are capable of, potentially leading to correlations of rIIV with the mean of the same or related traits (Biro et al., 2018). There are often limitations in the availability and/or reliability of information on the current environment (DeWitt et al., 1998), and this lack of certainty could increase rIIV. If individuals differ in their ability or propensity to accrue and compute information, for example, due to exploration tendencies or sensory system variation, this would lead to variation in rIIV and covariances with that trait. Under such a scenario, individuals that are more plastic would also exhibit greater residual variance as they respond not directly to the environmental state, but rather to their perception of the environmental state.

### 3.3 Correlation of rIIV across traits

Where multiple traits were observed from the same sample of individuals, we ran analyses with a multivariate DHGLM, which was possible for 19 correlation estimates from 8 studies. This allowed us to assess whether animals were generally (un)predictable across multiple traits. Such correlations are predicted to arise if residual variance represents instabilities in underlying latent state variables, such as physiological or informational state (Stamps & Frankenhuis, 2016). Alternatively, such correlations could simply arise when two traits are strongly or directly linked, as a change in expression of one trait would necessitate a change in expression of the other. For instance, datasets considered here include multiple different metabolic traits from the same individuals (Careau, Hoye, et al., 2014; Norin & Gamperl, 2017; Velasque & Briffa, 2016), and variance in aerobic systems will thus create variance across these related traits. Likewise, mathematical linkages between the visual orientation and flight initiation distance assays analyzed from Allan et al. (2020) led to a strong rIIV‐rIIV correlations among individuals. Analogous to behavioral plasticity across multiple traits (Mitchell & Houslay, 2021), discussions of among‐individual correlations in predictability need to be placed in the context of other trait correlations, rather than being discussed separately as a “predictability syndrome” (*sensu* Hertel, Royauté, et al., 2020; O'Dea et al., 2020).

Here, we extracted the among‐individual correlations of intercepts of the pairs of traits and the correlation in rIIV of the same trait. As we were interested in the degree to which the means were correlated (not the direction of the correlation), we used the *r* ^2^ value of mean intercept correlations, which was logit‐transformed. The model was run as a multivariate meta‐analysis, estimating the mean correlation among traits of rIIV and the correlation among estimates with the *r* ^2^ of the intercepts. There was an overall positive correlation across traits in rIIV, though the effect was very small and overlapped 0 (*r* = 0.138 [−0.02, 0.29]). This means that individuals that were predictable in one trait tended to also be predictable in other traits. However, data were limited with just 19 estimates from 8 studies, and many of these estimates were very imprecise (as reflected by the large 95% CRI) and therefore largely uninformative to the model. Estimates also came from highly heterogeneous studies, and are therefore hard to generalize and should be treated as indicative only.

There was also the predicted positive correlation between the among‐individual correlation in rIIV and the *r* ^2^ of the mean intercepts, indicating that when traits are strongly linked, this extends to rIIV. While this effect also overlapped with 0 (*r* = 0.567 [−0.042, 0.94])—reflecting a lack of power—the effect is likely real. Had we have instead evaluated the residual correlation (rather than among‐individual correlations), the effect would be partially underpinned by mathematical dependencies. A more suitable null hypothesis when traits are strongly linked would therefore be a perfect correlation of rIIV across traits (i.e., *r* = 1). Quantifying the residual correlation was not possible as some of our (already limited) number of relevant datasets were not measured concurrently in time. Further, existing methods to estimate the residual covariance in a DHGLM assume a constant correlation coefficient, while the variances are changing. The covariance (and slope) therefore scale with the variances to maintain a constant correlation coefficient—an assumption that is biologically unrealistic and likely to yield spurious results.

### 3.4 Causes of individual variation in rIIV in physiological traits

The existence of individual variation in rIIV has many potential consequences to the fitness of an animal. The evolution of homeostasis is a clear example of directional selection reducing rIIV (Cannon, 1929), whereby feedback mechanisms have evolved to set an upper and lower bound of a trait, thus limiting variation. Such examples include the feedback between agonistic and antagonistic hormones, which rapidly return an animal to homeostatic bounds after a perturbation from that state (Romero et al., 2009). Similar behavioral and metabolic reactions exist in homeotherms to maintain core body temperature within a narrow temperature range (Bligh, 1979), and such variation in internal stability can have clear and intuitive proximate constraints such as surface area to volume ratios, which insulate the body from large temperature swings, most apparent among taxa (Gordon, 2012). This variation in internal state will then create variation in other linked physiological or behavioral traits. Thus, we should predict greater rIIV in endotherms' physiology and behavior when held outside of their thermal neutral zone, even if the environmental temperature is highly stable.

While it is initially most intuitive to think of the effect physiology will have on behavioral variation, behavior will also affect physiological variation. Continuing with the example of homeothermy, while a change in body temperature can cause a behavioral response (e.g., shivering), activity will also increase the body temperature (or increase O_2_ consumption). Therefore, behavior will affect measurements of physiological and performance traits. Many physiological measures require an animal to reach a peak capability, such example datasets may include maximum metabolic rate (Careau, Hoye, et al., 2014; Norin & Gamperl, 2017) or burst sprint speed (Adolph & Pickering, 2008). Similarly, in measuring resting and basal metabolic rate or baseline endocrine levels, the ability of an individual to reach this minimum will also be affected by behavior, such as an individual's ability to acclimate to, and rest in a respirometry chamber (Biro et al., 2016; Jäger et al., 2017). The effect of such factors could generate individual differences in rIIV, if animals are differentially sensitive or motivated to reach these states. Where there is a general lack of control, these factors could also reduce heterogeneity by adding uniformly to the mean residual variance.

## 4 CONCLUSION

Individual variation in residual variance was almost ubiquitous across a wide range of taxa and traits considered in this paper. However, the extent of variation in rIIV was highly variable across the different datasets, as was the relationship of individual differences in rIIV to trait means. Contrary to predictions, heterogeneous residual variance did not appear to occur due to a lack of control of environmental factors—in contrast, laboratory studies showing a higher CV_P_ on average than field studies. Together with work now reporting heritability in behavioral predictability—we argue that rIIV should be viewed as part of a broader trait structure. While interest has continued to grow in individual variation in behavior and physiology, rIIV opens the door to a better understanding of the maintenance of individual variation. Studies often use repeatability estimates as a measure of the extent to individual variation, proportional to the residual variation. The study of rIIV will better allow us to explore the ecological factors and proximate constraints that promote or erode trait variance at these different hierarchical levels. Studies are now needed to better integrate residual variation into the broader phenotype, by linking the causes of this variability to underlying proximate causes—be they physiological, neurological, or genetic. In parallel, studies are also required to test the predictions of functional (un)predictability, with studies which may tie rIIV to individual fitness.

## CONFLICT OF INTEREST

None declared.

## AUTHOR CONTRIBUTIONS

**David J. Mitchell:** Conceptualization (equal); Data curation (lead); Formal analysis (lead); Visualization (lead); Writing‐original draft (lead); Writing‐review & editing (equal). **Christa Beckmann:** Conceptualization (equal); Supervision (supporting); Writing‐review & editing (equal). **Peter A. Biro:** Conceptualization (equal); Formal analysis (supporting); Funding acquisition (lead); Investigation (equal); Supervision (lead); Writing‐review & editing (equal).

## Supporting information

Appendix S1

## Data Availability

Detailed analysis code and output of models run, and the summary statistics underlying the meta‐analytic component can be found on the open science framework: (Mitchell, 2021): URL https://osf.io/znqra/.
