# Supplementary material for: Understanding the unexplained: The magnitude and correlates of individual differences in residual variance
Source: Ecol Evol. 2021 May 3;11(12):7201–10. doi: 10.1002/ece3.7603 (PMC8216950; doi:10.1002/ece3.7603)

Meta-analyses of residual variance

David J. Mitchell

24-Mar-2021

Sumary of dataset

First, just a couple summary. We have 64 estimates from 39 studies. These come from 25 different species.

Here's a list of the common names of the animals:

```
##  [1] "Agile.frog.tadpole"    "Baboon"                "Brown.bear"            "Chipmunk"
##  [5] "Cunner"                "Damselfish"            "Dog"                   "Elephant"
##  [9] "Fallow.deer"           "Guppy"                 "Hermit.Crab"           "Mice"
## [13] "Mosquitofish"          "Namibian.rock.agama"   "Periwinkle"            "Pied.flycatcher"
## [17] "Red-winged.Blackbird"  "Sea.anemone"           "Shore.crab"            "Slater"
## [21] "Sparrow"               "Stickleback"           "Wester.fence.lizard"   "Yellow-footed.marmots"
## [25] "Zebrafish"
```

Guppies are the best represented species with 16 estimates from 7 studies, and four independent lab groups. Next are the hermit crabs, all of which are from Mark Briffa's lab, with 9 estimates from 6 studies.

Here is a list of citations:

```
##  [1] "Adolph and Pickering 2008 JExpBiol"      "Allan et al 2020 SciAdv"
##  [3] "Biro and Adriaenssens 2013 AmNat"        "Biro et al 2010 ProcB"
##  [5] "Biro et al 2016 EcolEvol"                "Bridger et al 2015 ProcB"
##  [7] "Briffa 2013 BiolLett"                    "Briffa et al 2013 AnimBehav"
##  [9] "Careau et al 2015 JExpBiol"              "Cornwell et al 2018 JAnimEcol"
## [11] "Cornwell et al 2020 AnimBehav"           "Eisenmann et al 2009 EurJApplPhysiol"
## [13] "Furtbauer et al 2015 FunctEcol"          "Furthauer 2015 RSOS"
## [15] "Goold and Newberry 2016 RSOS"            "Herczeg et al 2019 JEvolBiol"
```

```
## [17] "Hertel et al 2020 JAnimEcol"             "Hertel et al 2020 MovEcol"
## [19] "Highcock and Carter 2014 PLoSOne"        "Horvath et al 2019 SciTotEnv"
## [21] "Jennings et al 2013 AnimBehav"           "Jolles et al 2019 AnimBehav"
## [23] "Kurvers et al 2018 SciRep"               "Martin et al 2018 JEvolBiol"
## [25] "Mitchell and Biro 2017 ProcB"            "Mitchell et al 2016 RSOS"
## [27] "Mitchell et al 2020 EcoEvoRxiv"          "Mitchell et al 2020b EcoEvoRxiv"
## [29] "Montiglio et al 2015 BehavEcolSociobiol" "Nanninga et al 2020 JHazMatLett"
## [31] "Norin and Gamberl 2016 FunctEcol"        "O'Dea et al 2020 EcoEvoRxiv"
## [33] "Osborn and Briffa 2017 AnimBehav"        "Prentice et al 2020 JEvolBiol"
## [35] "Stamps et al 2012 AnimBehav"             "Urszan et al 2018 JAnimEcol"
## [37] "Velasque and Briffa 2016 Behaviour"      "Westneat et al 2013 BehavEvol"
## [39] "Westneat et al 2017 BehavEvolSociobiol"
```

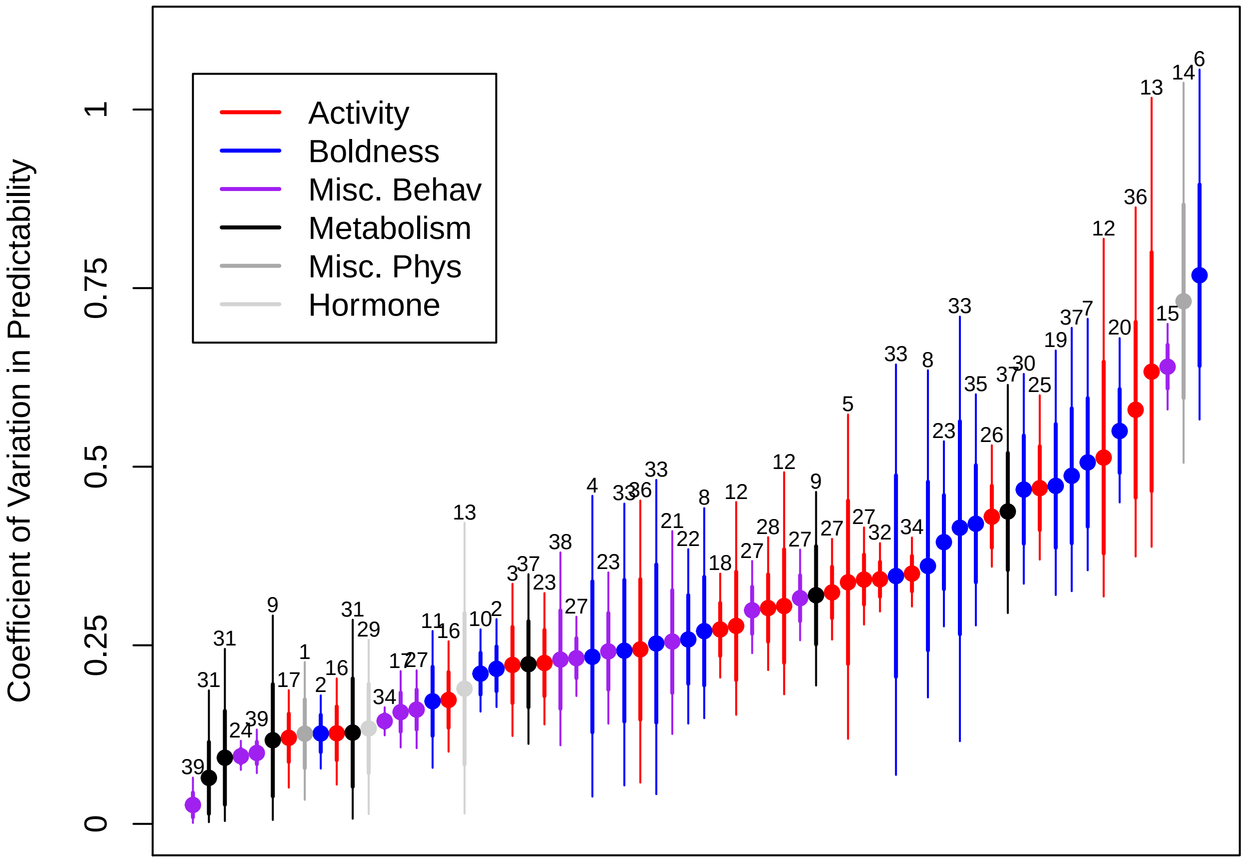

The best estimates (points) of CV_P show a little bit of a positive skew, as does the credible distrbiution on these estimates – as shown by the asymmetry of the 95% credible intervals. Log-transforming the best estimates does appear to improve normality. This also makes the the estimates in later models positive definite (i.e. $CV_P > 0$). However, log-transforming the credible distributions leads to a strong negative-skew, far worse than the existing positive-skew.

Funnel plot

To start, here are plots of the best estimates (log-transformed on the x-axis) against the sample size ($log(N_{observations})$) and against the precision as as given by:

$$\tau_i = \frac{1}{\sigma_{CV_i}}$$

As we can see, when plotted against the sample sizes, estimates seem fairly randomly distpersed, indicating we do not have a big confirmation bias or file-drawer effect. The plots against τ show larger estimates have lower precision, which is due to scaling and the lower bound of 0 truncating the estimate variance. This effect is observable in the simulations for Table 3 of Cleasby et al. (2014).

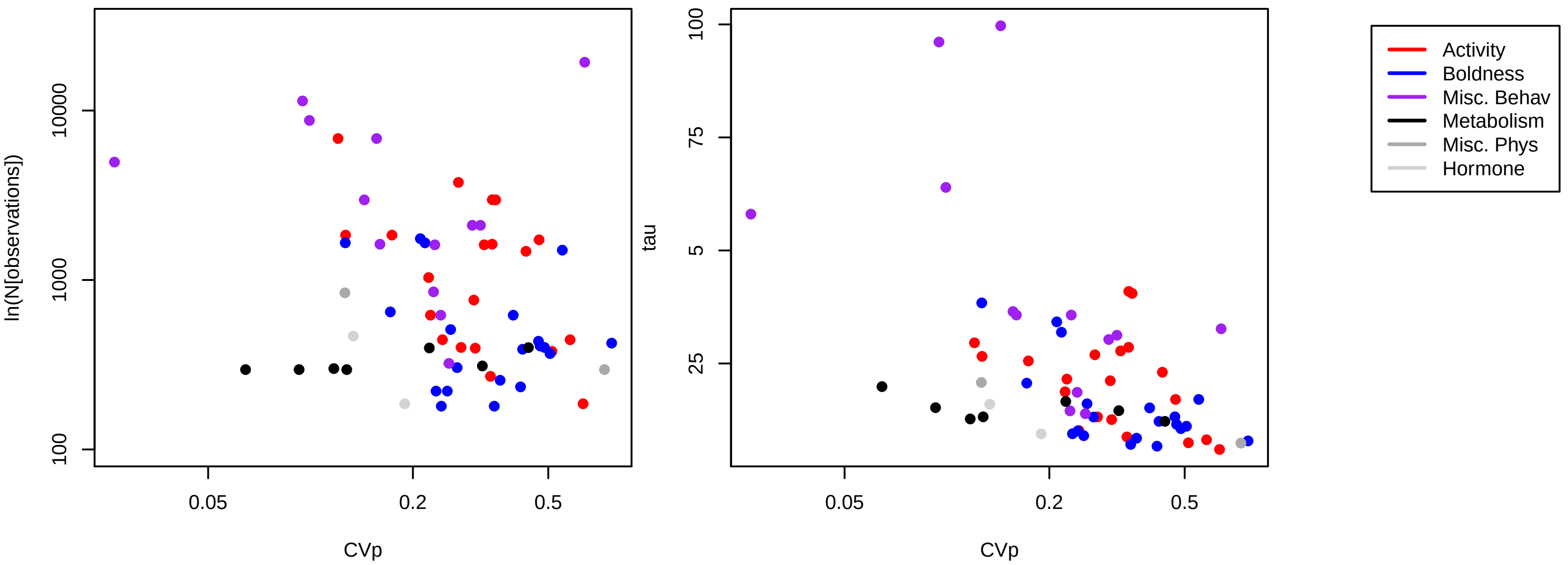

Overall CV_P

The first model simply calculates a weighted mean of all datasets. The models have two random intercepts. The first is just the observation number, which keeps an estimate as a level of replication. Some studies have multiple estimates due to having measured multiple traits, there are 9 studies that do this (Allan et al 2020 SciAdv, Careau et al 2015 JExpBiol, Furtbauer et al 2015 FunctEcol, Hertel et al 2020 JAnimEcol, Kurvers et al 2018 SciRep, Mitchell et al 2020 EcoEvoRxiv, Norin and Gamberl 2016 FunctEcol, Prentice et al 2020 JEvolBiol, Velasque and Briffa 2016 Behaviour, Westneat et al 2017 BehavEvolSociobiol). One other study reported variance in rIIV for four other traits (O'Dea et al 2020 EcoEvoRxiv), though due to large ceiling effects which truncate variance, these estimates were discarded. Others have multiple subpopulations or treatments within the study (Briffa et al 2013 AnimBehav, Eisenmann et al 2009 EurJApplPhysiol, Herczeg et al 2019 JEvolBiol, Mitchell et al 2020 EcoEvoRxiv, Urszan et al 2018 JAnimEcol).

Due to the skew among estimates, and because CV_P must be greater than 0, I have modelled CV_P on the log-scale. However, as the credible distribution on each estimate is closer to symmetry without transforming, I have retained the error variance around each estimate on the observed

scale. This means that for the simple first model calculating a weighted mean, a link-function is moved to the right-hand side of the equation. This is done through the non-linear commands in brms.

Where a model of transformed data would be

$$log(CV_i) = \beta_0 + obs_i + Study_j + \epsilon_i$$

$$obs_i = Norm(0, \sigma)$$

$$Study_j = Norm(0, \sigma)$$

$$\epsilon_i = Norm(0, \sigma)$$

where ϵ_i refers to the estimated precision in the estimate, i.e. `se(SD.CVp)`. This is shown by the bold line around the point above.

When returned to the observed scale, these variances become log-normal. Instead, the models below move the log-scaling to the righthand side, and omits the error variances (i.e. ϵ_i) from the link-function. This retain the precision estimates from the original scale. This can instead be written as:

$$CV_i = exp(\beta_0 + obs_i + Study_j) + \epsilon_i$$

Thus obs_i and $Study_j$ are log-normal, with the mean given by the fixed effects, while $\epsilon_k = Norm(0, \sigma)$.

```
ds$obs <- 1:length(ds$CVp)

mod1 <- bf(CVp|se(SD.CVp) ~ exp(y),
           y ~ 1 + (1|obs) + (1|Study), nl = T)

prior1 <- c(set_prior('normal(0,5)', class = 'b', nlpar = 'y'),
            set_prior('cauchy(0,5)', class = 'sd', nlpar = 'y'))

fit1 <- brm(mod1, data = ds, family = gaussian(), prior = prior1,
           warmup = 500, iter = 5000, chains = 4, cores = 4,
           control = list(adapt_delta = 0.97), seed = 42, file = 'model_runs/fit1')

print(summary(fit1), digits = 3)
```

```
##  Family: gaussian
##   Links: mu = identity; sigma = identity
## Formula: CVp | se(SD.CVp) ~ exp(y)
##          y ~ 1 + (1 | obs) + (1 | Study)
##    Data: ds (Number of observations: 64)
```

```
## Samples: 4 chains, each with iter = 5000; warmup = 500; thin = 1;
##          total post-warmup samples = 18000
##
## Group-Level Effects:
## ~obs (Number of levels: 64)
##                   Estimate Est.Error l-95% CI u-95% CI  Rhat Bulk_ESS Tail_ESS
## sd(y_Intercept)    0.369     0.085    0.235    0.563 1.001     3432     4437
##
## ~Study (Number of levels: 39)
##                   Estimate Est.Error l-95% CI u-95% CI  Rhat Bulk_ESS Tail_ESS
## sd(y_Intercept)    0.455     0.118    0.191    0.676 1.001     3551     2377
##
## Population-Level Effects:
##               Estimate Est.Error l-95% CI u-95% CI  Rhat Bulk_ESS Tail_ESS
## y_Intercept   -1.368     0.099   -1.563   -1.172 1.001    12603    11886
##
## Family Specific Parameters:
##         Estimate Est.Error l-95% CI u-95% CI  Rhat Bulk_ESS Tail_ESS
## sigma    0.000     0.000    0.000    0.000 1.000    18000    18000
##
## Samples were drawn using sampling(NUTS). For each parameter, Bulk_ESS
## and Tail_ESS are effective sample size measures, and Rhat is the potential
## scale reduction factor on split chains (at convergence, Rhat = 1).
```

This estimate can be returned to the observed scale:

```
summary(coda::as.mcmc(exp(as.matrix(fit1, pars = "b")[,1])))
```

```
##
## Iterations = 1:18000
## Thinning interval = 1
## Number of chains = 1
## Sample size per chain = 18000
##
## 1. Empirical mean and standard deviation for each variable,
##    plus standard error of the mean:
##
##           Mean             SD       Naive SE Time-series SE
```

```
##      0.2559319      0.0254970      0.0001900      0.0002257
##
## 2. Quantiles for each variable:
##
##   2.5%    25%    50%    75%  97.5%
## 0.2094 0.2384 0.2548 0.2718 0.3099
```

Physiology vs. behaviour

Here is the analysis splitting physiological and behaviour traits. Physiology is quite broadly defined here, with 2 endocrine studies (2 estimates), two metabolism studies (5 estimates), a haemolymph study (1 estimate) and also includes a performance study (1 estimate of sprint speed, Adolph and Pickering 2008 *JExpBiol*). I have also separated the variances in estimates by this grouping factor. Furtbauer et al 2015 *FunctEcol* record behaviour and CORT, while Velasque and Briffa 2016 *Behaviour* record behaviour and metabolism - so `Study` does not have separate variances.

```
mod2 <- bf(CVp|se(SD.CVp) ~ exp(y),
           y ~ 0 + Trait.Type + (1|Study) + (1|gr(obs, by = Trait.Type)), nl = T)

prior2 <- c(set_prior('normal(0,5)', class = 'b', nlpar = 'y'),
            set_prior('cauchy(0,5)', class = 'sd', nlpar = 'y'))


fit2 <- brm(mod2, data = ds, family = gaussian(), prior = prior2,
           warmup = 500, iter = 5000, chains = 4, cores = 4,
           control = list(adapt_delta = 0.97), seed = 42, file = 'model_runs/fit2')


print(summary(fit2), digits = 3)
```

```
##  Family: gaussian
##   Links: mu = identity; sigma = identity
## Formula: CVp | se(SD.CVp) ~ exp(y)
##          y ~ 0 + Trait.Type + (1 | Study) + (1 | gr(obs, by = Trait.Type))
##    Data: ds (Number of observations: 64)
## Samples: 4 chains, each with iter = 5000; warmup = 500; thin = 1;
##          total post-warmup samples = 18000
##
## Group-Level Effects:
## ~obs (Number of levels: 64)
```

```
##                                    Estimate Est.Error l-95% CI u-95% CI  Rhat Bulk_ESS Tail_ESS
## sd(y_Intercept:Trait.TypeBehaviour)     0.347     0.081    0.218    0.537 1.001     3619     3935
## sd(y_Intercept:Trait.TypePhysiology)    0.668     0.378    0.110    1.586 1.001     5055     6111
##
## ~Study (Number of levels: 39)
##                  Estimate Est.Error l-95% CI u-95% CI  Rhat Bulk_ESS Tail_ESS
## sd(y_Intercept)    0.458     0.115    0.201    0.675 1.001     3566     2265
##
## Population-Level Effects:
##                         Estimate Est.Error l-95% CI u-95% CI  Rhat Bulk_ESS Tail_ESS
## y_Trait.TypeBehaviour    -1.301     0.103   -1.506   -1.098 1.000    11812    11566
## y_Trait.TypePhysiology   -1.833     0.342   -2.607   -1.245 1.000    13648     9594
##
## Family Specific Parameters:
##       Estimate Est.Error l-95% CI u-95% CI  Rhat Bulk_ESS Tail_ESS
## sigma    0.000     0.000    0.000    0.000 1.000    18000    18000
##
## Samples were drawn using sampling(NUTS). For each parameter, Bulk_ESS
## and Tail_ESS are effective sample size measures, and Rhat is the potential
## scale reduction factor on split chains (at convergence, Rhat = 1).
```

```r
b <- exp(as.matrix(fit2, pars = "b"))[,1:2]
summary(coda::as.mcmc(b))
```

```
##
## Iterations = 1:18000
## Thinning interval = 1
## Number of chains = 1
## Sample size per chain = 18000
##
## 1. Empirical mean and standard deviation for each variable,
##    plus standard error of the mean:
##
##                          Mean      SD  Naive SE Time-series SE
## b_y_Trait.TypeBehaviour  0.2736 0.02831 0.0002110       0.000266
## b_y_Trait.TypePhysiology 0.1689 0.05507 0.0004105       0.000452
##
## 2. Quantiles for each variable:
```

```
##
##                               2.5%    25%    50%    75%  97.5%
## b_y_Trait.TypeBehaviour  0.22173 0.2542 0.2720 0.2913 0.3334
## b_y_Trait.TypePhysiology 0.07374 0.1325 0.1647 0.1999 0.2880
```

```
fit2b <- brm(mod2, data = subset(ds, Measure != 'Haemolymph.Density'),
             ds, family = gaussian(), prior = prior2,
             warmup = 500, iter = 5000, chains = 4, cores = 4,
             control = list(adapt_delta = 0.97), seed = 42, file = 'model_runs/fit2b')

b2 <- exp(as.matrix(fit2b, pars = "b"))[,1:2]
```

This means on average the physiological traits have a lower CV_P than the behavioural traits, though this is insignificant: -0.105 [-0.215, 0.022]. The haemolymph density study has a high CV_P, which is driving the lack of a difference. If we remove this observation, the difference is then significant: -0.127 [-0.220, -0.027].

We see in the next section that metabolism is lower, and the two hormone studies also have low CV_P.

Separated by trait

The following analysis breaks down traits into more detail. For 'activity' and 'boldness' we have a good number of estimates, disproportionately guppies for activity, and hermit crabs for boldness. Numerous studies end up getting pooled into "miscellaneous" as we just have one or two examples of that trait. For example, we only have 1 of haemolymph desity, aggression, parental provisioning, exploration and a few more. I have split these "miscelaneous" traits by whether they're physiological or behavioural.

I have kept the separate variances among estimates by splitting physiology and behaviour.

```
ds$Trait <- as.character(ds$Trait)
ds$Trait[ds$Trait == 'Hormone'] <- 'Miscellaneous'
ds$Trait <- as.character(factor(ds$Trait):ds$Trait.Type)

mod3 <- bf(CVp|se(SD.CVp) ~ exp(y),
           y ~ 0 + Trait + (1|Study) + (1|gr(obs, by = Trait.Type)), nl = T)

prior3 <- c(set_prior('normal(0,5)', class = 'b', nlpar = 'y'),
            set_prior('cauchy(0,5)', class = 'sd', nlpar = 'y'))


fit3 <- brm(mod3, data = ds, family = gaussian(), prior = prior3,
```

```r
          warmup = 500, iter = 5000, chains = 4, cores = 4,
          control = list(adapt_delta = 0.98), seed = 42, file = 'model_runs/fit3')


print(summary(fit3), digits = 3)
```

```
##  Family: gaussian
##   Links: mu = identity; sigma = identity
## Formula: CVp | se(SD.CVp) ~ exp(y)
##          y ~ 0 + Trait + (1 | Study) + (1 | gr(obs, by = Trait.Type))
##    Data: ds (Number of observations: 64)
## Samples: 4 chains, each with iter = 5000; warmup = 500; thin = 1;
##          total post-warmup samples = 18000
##
## Group-Level Effects:
## ~obs (Number of levels: 64)
##                                     Estimate Est.Error l-95% CI u-95% CI  Rhat Bulk_ESS Tail_ESS
## sd(y_Intercept:Trait.TypeBehaviour)     0.309     0.082    0.176    0.497 1.001     3463     4178
## sd(y_Intercept:Trait.TypePhysiology)    0.816     0.451    0.173    1.920 1.000     5884     6668
##
## ~Study (Number of levels: 39)
##                 Estimate Est.Error l-95% CI u-95% CI  Rhat Bulk_ESS Tail_ESS
## sd(y_Intercept)    0.424     0.109    0.183    0.634 1.001     3472     2907
##
## Population-Level Effects:
##                              Estimate Est.Error l-95% CI u-95% CI  Rhat Bulk_ESS Tail_ESS
## y_TraitActivity:Behaviour        -1.270     0.135   -1.535   -1.008 1.001    10180    11747
## y_TraitBoldness:Behaviour        -1.123     0.145   -1.413   -0.834 1.000    10241    12552
## y_TraitMetabolism:Physiology     -1.926     0.496   -3.061   -1.090 1.000     9681     8532
## y_TraitMiscellaneous:Behaviour   -1.674     0.160   -1.993   -1.362 1.000     9259    11020
## y_TraitMiscellaneous:Physiology  -1.741     0.571   -2.966   -0.695 1.000    10960     9386
##
## Family Specific Parameters:
##       Estimate Est.Error l-95% CI u-95% CI  Rhat Bulk_ESS Tail_ESS
## sigma    0.000     0.000    0.000    0.000 1.000    18000    18000
##
## Samples were drawn using sampling(NUTS). For each parameter, Bulk_ESS
## and Tail_ESS are effective sample size measures, and Rhat is the potential
```

```
## scale reduction factor on split chains (at convergence, Rhat = 1).
```

```r
b <- exp(as.matrix(fit3, pars = "b"))[,1:5]
summary(coda::as.mcmc(b))
```

```
##
## Iterations = 1:18000
## Thinning interval = 1
## Number of chains = 1
## Sample size per chain = 18000
##
## 1. Empirical mean and standard deviation for each variable,
##    plus standard error of the mean:
##
##                                      Mean      SD  Naive SE Time-series SE
## b_y_TraitActivity:Behaviour       0.2834 0.03826 0.0002852      0.0003775
## b_y_TraitBoldness:Behaviour       0.3287 0.04810 0.0003585      0.0004620
## b_y_TraitMetabolism:Physiology    0.1630 0.08207 0.0006117      0.0008343
## b_y_TraitMiscellaneous:Behaviour  0.1898 0.03046 0.0002270      0.0003131
## b_y_TraitMiscellaneous:Physiology 0.2051 0.13887 0.0010351      0.0013892
##
## 2. Quantiles for each variable:
##
##                                       2.5%    25%    50%    75%  97.5%
## b_y_TraitActivity:Behaviour       0.21550 0.2568 0.2811 0.3075 0.3649
## b_y_TraitBoldness:Behaviour       0.24346 0.2955 0.3252 0.3581 0.4344
## b_y_TraitMetabolism:Physiology    0.04683 0.1119 0.1524 0.2005 0.3364
## b_y_TraitMiscellaneous:Behaviour  0.13633 0.1688 0.1875 0.2085 0.2562
## b_y_TraitMiscellaneous:Physiology 0.05150 0.1281 0.1812 0.2473 0.4993
```

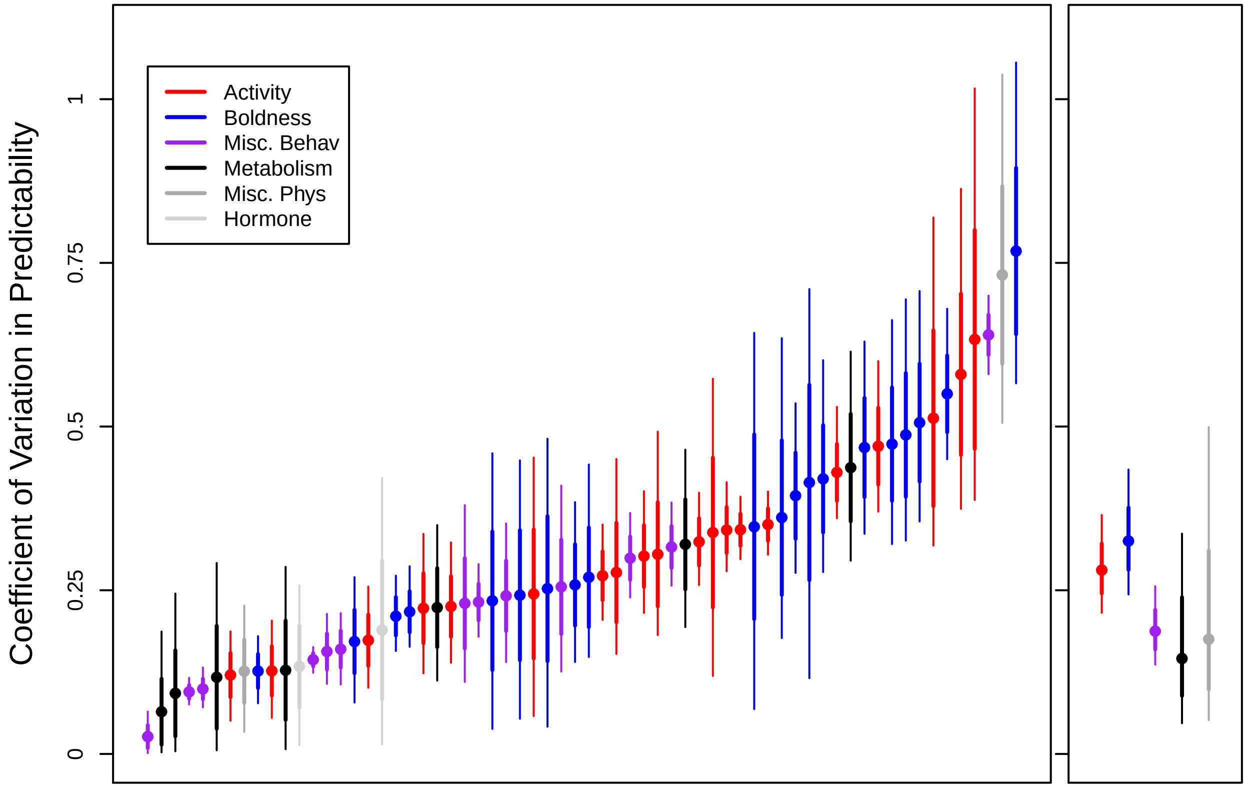

Field vs. laboratory

Finally, the comparison of lab and field studies. A number of people have posited that individuals may differ in rIIV due to the lack of control of the environment. The argument being that individuals commonly vary in contextual plasticity, and this could give rise to heterogeneous residual variance, or some individuals might be assayed under more variable conditions. The analysis here statistically controls for the differences among trait types, and retains the variance differences.

As you can see, the opposite is actually true, which we posit in the main text is due to the lack of control swamping out intrinsic individual differences in residual variance, i.e. differences in variability get lost when extra noise is added. This result does require a pinch of salt – the estimates in the field tend to commonly be "miscellaneous" traits, boldness in the field is flight initiation distance rather than latency to emerge, activity in the field is over much larger spatial scales than laboratory assays in an open field. There are therefore likely confounds which affect this result in unknown ways.

```r
mod4 <- bf(CVp|se(SD.CVp) ~ exp(y),
           y ~ Trait + Field + (1|Study) +
             (1|gr(obs, by = Trait.Type)), nl = T)

prior4 <- c(set_prior('normal(0,5)', class = 'b', nlpar = 'y'),
            set_prior('cauchy(0,5)', class = 'sd', nlpar = 'y'))


fit4 <- brm(mod4, data = ds, family = gaussian(), prior = prior4,
           warmup = 500, iter = 5000, chains = 4, cores = 4,
           control = list(adapt_delta = 0.97), seed = 42, file = 'model_runs/fit4')

print(summary(fit4), digits = 3)
```

```
##  Family: gaussian
##   Links: mu = identity; sigma = identity
## Formula: CVp | se(SD.CVp) ~ exp(y)
##          y ~ Trait + Field + (1 | Study) + (1 | gr(obs, by = Trait.Type))
##    Data: ds (Number of observations: 64)
## Samples: 4 chains, each with iter = 5000; warmup = 500; thin = 1;
##          total post-warmup samples = 18000
##
## Group-Level Effects:
## ~obs (Number of levels: 64)
##                                    Estimate Est.Error l-95% CI u-95% CI  Rhat Bulk_ESS Tail_ESS
## sd(y_Intercept:Trait.TypeBehaviour)     0.328     0.080    0.189    0.497 1.003     2328     6755
## sd(y_Intercept:Trait.TypePhysiology)    0.732     0.364    0.204    1.584 1.000     4772     5182
##
## ~Study (Number of levels: 39)
##                 Estimate Est.Error l-95% CI u-95% CI  Rhat Bulk_ESS Tail_ESS
## sd(y_Intercept)    0.306     0.127    0.034    0.532 1.005     1456     2453
##
## Population-Level Effects:
##                      Estimate Est.Error l-95% CI u-95% CI  Rhat Bulk_ESS Tail_ESS
## y_Intercept            -1.164     0.126   -1.414   -0.915 1.000     8963    12051
## y_TraitBoldness         0.135     0.171   -0.200    0.472 1.000    10201    11873
## y_TraitMetabolism      -0.720     0.450   -1.720    0.071 1.001     9920    10139
## y_TraitMiscellaneous   -0.295     0.163   -0.611    0.034 1.000     9210    10408
```

```
## y_FieldY               -0.574     0.200    -0.962    -0.168 1.000    11396    12762
##
## Family Specific Parameters:
##       Estimate Est.Error l-95% CI u-95% CI  Rhat Bulk_ESS Tail_ESS
## sigma    0.000     0.000    0.000    0.000 1.000    18000    18000
##
## Samples were drawn using sampling(NUTS). For each parameter, Bulk_ESS
## and Tail_ESS are effective sample size measures, and Rhat is the potential
## scale reduction factor on split chains (at convergence, Rhat = 1).
```

Publication bias

While at the beginning of this document I plotted out effect sizes vs. sample sizes and estimate precision, another way we may evaluate the potential reporting biases is to compare the studies which had (n = 39) and had not (n = 25) considered individual differences in residual variance. If these patterns were frequently identified through initial data exploration and visualisation, or simply not reported where the results came out to be null, then this would be evident when comparing the reported CV_P estimates of those which did not consider individual variation in rIIV.

```
mod1b <- bf(CVp|se(SD.CVp) ~ exp(y),
           y ~ Predictability.Considered + (1|Study) +
             (1|obs), nl = T)

mod4b <- bf(CVp|se(SD.CVp) ~ exp(y),
           y ~ Trait + Field + Predictability.Considered + (1|Study) +
             (1|gr(obs, by = Trait.Type)), nl = T)

prior1b <- c(set_prior('normal(0,5)', class = 'b', nlpar = 'y'),
             set_prior('cauchy(0,5)', class = 'sd', nlpar = 'y'))

fit1b <- brm(mod1b, data = ds, family = gaussian(), prior = prior1b,
             warmup = 500, iter = 5000, chains = 4, cores = 4,
             control = list(adapt_delta = 0.97), seed = 42, file = 'model_runs/fit1b')

print(summary(fit1b), digits = 3)
```

```
##  Family: gaussian
##   Links: mu = identity; sigma = identity
## Formula: CVp | se(SD.CVp) ~ exp(y)
##          y ~ Predictability.Considered + (1 | Study) + (1 | obs)
```

```
##    Data: ds (Number of observations: 64)
## Samples: 4 chains, each with iter = 5000; warmup = 500; thin = 1;
##          total post-warmup samples = 18000
##
## Group-Level Effects:
## ~obs (Number of levels: 64)
##                 Estimate Est.Error l-95% CI u-95% CI  Rhat Bulk_ESS Tail_ESS
## sd(y_Intercept)    0.368     0.083    0.236    0.557 1.001     3798     4610
##
## ~Study (Number of levels: 39)
##                 Estimate Est.Error l-95% CI u-95% CI  Rhat Bulk_ESS Tail_ESS
## sd(y_Intercept)    0.457     0.117    0.199    0.678 1.001     3680     3051
##
## Population-Level Effects:
##                              Estimate Est.Error l-95% CI u-95% CI  Rhat Bulk_ESS Tail_ESS
## y_Intercept                    -1.497     0.164   -1.819   -1.172 1.000    11881    12223
## y_Predictability.ConsideredY    0.202     0.203   -0.197    0.602 1.000    11466    12218
##
## Family Specific Parameters:
##       Estimate Est.Error l-95% CI u-95% CI  Rhat Bulk_ESS Tail_ESS
## sigma    0.000     0.000    0.000    0.000 1.000    18000    18000
##
## Samples were drawn using sampling(NUTS). For each parameter, Bulk_ESS
## and Tail_ESS are effective sample size measures, and Rhat is the potential
## scale reduction factor on split chains (at convergence, Rhat = 1).
```

```
fit4b <- brm(mod4, data = ds, family = gaussian(), prior = prior1b, #same priors as 1b
             warmup = 500, iter = 5000, chains = 4, cores = 4,
             control = list(adapt_delta = 0.97), seed = 42, file = 'model_runs/fit4b')

print(summary(fit4b), digits = 3)
```

```
##  Family: gaussian
##   Links: mu = identity; sigma = identity
## Formula: CVp | se(SD.CVp) ~ exp(y)
##          y ~ Trait + Predictability.Considered + (1 | Study) + (1 | gr(obs, by = Trait.Type))
##    Data: ds (Number of observations: 64)
## Samples: 4 chains, each with iter = 5000; warmup = 500; thin = 1;
```

```
##          total post-warmup samples = 18000
##
## Group-Level Effects:
## ~obs (Number of levels: 64)
##                                      Estimate Est.Error l-95% CI u-95% CI  Rhat Bulk_ESS Tail_ESS
## sd(y_Intercept:Trait.TypeBehaviour)     0.302     0.081    0.171    0.486 1.000     3584     4311
## sd(y_Intercept:Trait.TypePhysiology)    0.799     0.433    0.169    1.883 1.000     5866     6475
##
## ~Study (Number of levels: 39)
##                  Estimate Est.Error l-95% CI u-95% CI  Rhat Bulk_ESS Tail_ESS
## sd(y_Intercept)    0.420     0.109    0.183    0.629 1.001     3319     2739
##
## Population-Level Effects:
##                              Estimate Est.Error l-95% CI u-95% CI  Rhat Bulk_ESS Tail_ESS
## y_Intercept                    -1.412     0.183   -1.781   -1.054 1.000    10353    11567
## y_TraitBoldness                 0.122     0.192   -0.256    0.499 1.000    11669    13471
## y_TraitHormone                 -1.786     1.922   -7.369    0.369 1.000     6112     5183
## y_TraitMetabolism              -0.600     0.499   -1.709    0.287 1.000    11171    11788
## y_TraitMiscellaneous           -0.417     0.171   -0.761   -0.089 1.000     9020    11412
## y_Predictability.ConsideredY    0.234     0.201   -0.163    0.627 1.000    11378    12546
##
## Family Specific Parameters:
##       Estimate Est.Error l-95% CI u-95% CI  Rhat Bulk_ESS Tail_ESS
## sigma    0.000     0.000    0.000    0.000 1.000    18000    18000
##
## Samples were drawn using sampling(NUTS). For each parameter, Bulk_ESS
## and Tail_ESS are effective sample size measures, and Rhat is the potential
## scale reduction factor on split chains (at convergence, Rhat = 1).
```

Correlation with the mean

Next are the analyses of the generality of an association between the mean expression of a trait and rIIV. Only boldness and activity rates have a sufficient number of studies to be informative, and the "miscellaneous" traits are too variable in their significance to be informative.

First, I am going to transform the correlation coefficients to Fisher's Z-correlation; $z = \frac{1}{2} * ln(\frac{1+\rho}{1-\rho})$. This is done for the upper and lower bound of the credible distribution, then I re-calculate the best estimate as halfway between those two values. The 95%CRI correspond to 1.96 standard deviations from the mean, so precision is calculated as $\sigma = \frac{\rho_{upper} - \rho_{lower}}{3.92}$.

The inverse of the Fisher's Z-correlation transform is then given as $\rho = \frac{exp(2z-1)}{exp(2z+1)}$ for the posterior.

```
Z.corr  <- function(r) {.5 * log((1+r)/(1-r))}
inv.Z   <- function(z) {(exp(2*z)-1) / (exp(2*z)+1)}

ds$LZ   <- Z.corr(ds$LowCorr)
ds$UZ   <- Z.corr(ds$UpCorr)
ds$mu.Z <- (ds$UZ + ds$LZ) / 2
ds$SD.Z <- (ds$UZ - ds$LZ) / 3.92
```

Boldness

All of the boldness measures here have bolder animals having lower values, except for Jolles et al. 2018, i.e. animals with longer latency to emerge or flight initiation distances are considered "shyer". I have multiplied the correlations by -1 to make positive correlations equate to bolder animals being more variable. We have 20 observations of boldness, though one study did not have an estimate for the correlation coefficient and the raw data was not available.

```
BOLD <- subset(ds, Trait == 'Boldness')
BOLD$mu.Z[BOLD$Study != 'Jolles_et_al-2019-AnimBehav'] <-
  BOLD$mu.Z[BOLD$Study != 'Jolles_et_al-2019-AnimBehav'] * -1

mod6 <- bf(mu.Z|se(SD.Z) ~ 1 + (1|obs))

prior6 <- c(set_prior('normal(0,5)', class = 'Intercept'),
            set_prior('cauchy(0,5)', class = 'sd'))


fit6 <- brm(mod6, data = BOLD, family = gaussian(), prior = prior6,
           warmup = 500, iter = 5000, chains = 4, cores = 4,
           control = list(adapt_delta = 0.97), seed = 42, file = 'model_runs/fit6')

r <- inv.Z(as.matrix(fit6, pars = "b")[,1])

print(summary(fit6), digits = 3)
```

```
##  Family: gaussian
##   Links: mu = identity; sigma = identity
## Formula: mu.Z | se(SD.Z) ~ 1 + (1 | obs)
##    Data: BOLD (Number of observations: 19)
```

```
## Samples: 4 chains, each with iter = 5000; warmup = 500; thin = 1;
##          total post-warmup samples = 18000
##
## Group-Level Effects:
## ~obs (Number of levels: 19)
##               Estimate Est.Error l-95% CI u-95% CI  Rhat Bulk_ESS Tail_ESS
## sd(Intercept)    0.474     0.120    0.278    0.749 1.000     4318     7850
##
## Population-Level Effects:
##           Estimate Est.Error l-95% CI u-95% CI  Rhat Bulk_ESS Tail_ESS
## Intercept    0.244     0.129   -0.007    0.506 1.001     4054     6019
##
## Family Specific Parameters:
##       Estimate Est.Error l-95% CI u-95% CI  Rhat Bulk_ESS Tail_ESS
## sigma    0.000     0.000    0.000    0.000 1.000    18000    18000
##
## Samples were drawn using sampling(NUTS). For each parameter, Bulk_ESS
## and Tail_ESS are effective sample size measures, and Rhat is the potential
## scale reduction factor on split chains (at convergence, Rhat = 1).
```

Returned from Z-scale to correlation coefficients, r = 0.236 [-0.007, 0.467]. There's lots of heterogeneity within these studies as seen by the variance in obs. Even within the hermit crab data, which makes up 9 estimates, correlation values range from -0.773 to 0.626.

Activity

For activity, we have 20 estimates, ranging from r = -0.719 to 0.480.

```
ACT <- subset(ds, Trait == 'Activity')

mod7 <- bf(mu.Z|se(SD.Z) ~ 1 + (1|obs))

prior7 <- c(set_prior('normal(0,5)', class = 'Intercept'),
            set_prior('cauchy(0,5)', class = 'sd'))

fit7 <- brm(mod7, data = ACT, family = gaussian(), prior = prior7,
           warmup = 500, iter = 5000, chains = 4, cores = 4,
           control = list(adapt_delta = 0.97), seed = 42, file = 'model_runs/fit7')
```

```r
r <- inv.Z(as.matrix(fit7, pars = "b")[,1])

print(summary(fit7), digits = 3)
```

```
##  Family: gaussian 
##   Links: mu = identity; sigma = identity 
## Formula: mu.Z | se(SD.Z) ~ 1 + (1 | obs) 
##    Data: ACT (Number of observations: 20) 
## Samples: 4 chains, each with iter = 5000; warmup = 500; thin = 1;
##          total post-warmup samples = 18000
## 
## Group-Level Effects: 
## ~obs (Number of levels: 20) 
##               Estimate Est.Error l-95% CI u-95% CI  Rhat Bulk_ESS Tail_ESS
## sd(Intercept)    0.383     0.095    0.231    0.602 1.000     4636     7586
## 
## Population-Level Effects: 
##           Estimate Est.Error l-95% CI u-95% CI  Rhat Bulk_ESS Tail_ESS
## Intercept   -0.152     0.101   -0.357    0.046 1.001     4183     6282
## 
## Family Specific Parameters: 
##       Estimate Est.Error l-95% CI u-95% CI  Rhat Bulk_ESS Tail_ESS
## sigma    0.000     0.000    0.000    0.000 1.000    18000    18000
## 
## Samples were drawn using sampling(NUTS). For each parameter, Bulk_ESS
## and Tail_ESS are effective sample size measures, and Rhat is the potential
## scale reduction factor on split chains (at convergence, Rhat = 1).
```

This gives a best estimate r of -0.149 [-0.342, 0.046]. So there's no indication that there's a meaningful correlation on average.

```r
IIVcorr <- subset(ds, is.na(ds$r_IIV) == F)
```

Correlation of rIIV among-traits

In the analyses of the raw data, where multiple traits were available for the same set of individuals, we ran models as multivariate. This therefore assessed the among-individual correlation in rIIV – thus assessing assess whether individuals are generally (un/)predictable across multiple traits. Where traits are functionally linked in the analyses (for instance Allan et al. 2020 provide data on visual orientation distance and flight initiation

distance), correlations at the among-individual level of means and residual variances will be very high. Therefore correlations in predictability are likely to be driven simply by this correlation. Residual correlations themselves were not assessable for all pairs of traits, and are problematic to estimate as they assume a constant correlation coefficient while the variances are changing. Instead we extracted among-individual correlations in mean behaviour from all such data. The direction of the correlation in means is irrelevant for the proposed functional link, so here we use the $logit(r^2)$ as an estimate of the extent to the correlation in means. Correlations in rIIV are predicted to be positive, so we again use the Z-correlation. Data is limited here with just 19 estimates from 8 studies. For many studies, there was little power to assess this correlation and the estimates therefore carry very little weight in the model. We have fit this model as a multivariate model to assess 1) the mean correlation of rIIV between different traits and 2) whether the extent of a correlation in means predicts the correlation in rIIV.

```
IIVcorr <- subset(ds, is.na(ds$r_IIV) == F)

IIVcorr$Lr   <- Z.corr(IIVcorr$Low_r_IIV)
IIVcorr$Ur   <- Z.corr(IIVcorr$Up_r_IIV)
IIVcorr$mu.r <- (IIVcorr$Ur + IIVcorr$Lr) / 2
IIVcorr$SD.r <- (IIVcorr$Ur - IIVcorr$Lr) / 3.92

logit <- function(p) {log(p - (1-p))}
IIVcorr$Lr2     <- Z.corr(IIVcorr$Low_r2_ints)
IIVcorr$Ur2     <- Z.corr(IIVcorr$Up_r2_ints)
IIVcorr$mu.synd <- (IIVcorr$Ur2 + IIVcorr$Lr2) / 2
IIVcorr$SD.synd <- (IIVcorr$Ur2 - IIVcorr$Lr2) / 3.92


mod8 <- bf(mu.r|se(SD.r) ~ 1 + (1|2|obs)) +
        bf(mu.synd|se(SD.synd) ~ 1 + (1|2|obs)) +
         set_rescor(F)

prior8 <- c(set_prior('normal(0,5)', class = 'Intercept', resp = c('mur', 'musynd')),
            set_prior('cauchy(0,5)', class = 'sd', resp = c('mur', 'musynd')),
            set_prior('lkj(1)', class = 'cor'))

fit8 <- brm(mod8, data = IIVcorr, family = gaussian(), prior = prior8,
           warmup = 500, iter = 5000, chains = 4, cores = 4,
           control = list(adapt_delta = 0.98), seed = 42, file = 'model_runs/fit8')

r <- inv.Z(as.matrix(fit8, pars = "b")[,1])

print(summary(fit8), digits = 3)
```

```
##  Family: MV(gaussian, gaussian)
##   Links: mu = identity; sigma = identity
##          mu = identity; sigma = identity
## Formula: mu.r | se(SD.r) ~ 1 + (1 | 2 | obs)
##          mu.synd | se(SD.synd) ~ 1 + (1 | 2 | obs)
##    Data: IIVcorr (Number of observations: 19)
## Samples: 4 chains, each with iter = 5000; warmup = 500; thin = 1;
##          total post-warmup samples = 18000
##
## Group-Level Effects:
## ~obs (Number of levels: 19)
##                                      Estimate Est.Error l-95% CI u-95% CI  Rhat Bulk_ESS Tail_ESS
## sd(mur_Intercept)                       0.257     0.082    0.116    0.440 1.000     8649     9193
## sd(musynd_Intercept)                    0.303     0.089    0.155    0.506 1.000     4650     7256
## cor(mur_Intercept,musynd_Intercept)    0.567     0.258   -0.042    0.935 1.000     3982     6659
##
## Population-Level Effects:
##                  Estimate Est.Error l-95% CI u-95% CI  Rhat Bulk_ESS Tail_ESS
## mur_Intercept       0.140     0.081   -0.020    0.301 1.001     8372    11219
## musynd_Intercept    0.255     0.078    0.108    0.419 1.001     8174    10720
##
## Family Specific Parameters:
##              Estimate Est.Error l-95% CI u-95% CI  Rhat Bulk_ESS Tail_ESS
## sigma_mur       0.000     0.000    0.000    0.000 1.000    18000    18000
## sigma_musynd    0.000     0.000    0.000    0.000 1.000    18000    18000
##
## Samples were drawn using sampling(NUTS). For each parameter, Bulk_ESS
## and Tail_ESS are effective sample size measures, and Rhat is the potential
## scale reduction factor on split chains (at convergence, Rhat = 1).
```

Overall there is a positive but negligible correlation of rIIV across traits, which when returned from the Z-scale gives a best estimate r of 0.138 [-0.020, 0.292]. Again, this effect needs a large pinch of salt that there are few studies which are highly heterogenous in nature and a few particularly powerful studies likely drive the effect/absence of effect. There's also an indication that when the correlation between the means of traits is strong, this correlates with a positive correlation between rIIV between those same traits. This effect was insignificant, though the effect is very likely to be real. Had we have been able to examine the residual correlation instead of the among-individual correlation, these correlations would be largely mathematically linked.

Simulation of $CV[P]$

Here, I will present the code which created the simulations that went into Figure 2. Firstly, I have created a simple simulation with all individual means fixed to 0. I have restandardised the estimates throughout so that they are centred on 0 and the variances among BLUPs are re-standardised to be what they are meant to be – i.e. they're not perturbed from these values by random chance. The individuals in the simulation are then ranked based on the on the observed data, not the values that they are simulated from. This means that even for the null scenario, there appears to be some fanning of variance from left to right.

```
simulate <- function(CVp, nID = 30, nrep = 20, seed = 42){
  set.seed(seed)
  ID    <- rnorm(nID,0,1)
  ID    <- (ID - mean(ID))/sd(ID)

  ID1   <- ID*sqrt(log(CVp^2 + 1))
  ds1   <- data.frame( id = sort(rep(c(1:nID),nrep)), sd = sort(rep(exp(ID1),nrep)),
                     rep = (rep(c(1:nrep),nID)))
  ds1$y <- rnorm(nID*nrep, 0, ds1$sd)

  df <- aggregate(data = ds1, y ~ id, FUN = mean)
  df$sd.id <- aggregate(data = ds1, y ~ id, FUN = sd)$y
  names(df)[2] <- "mean"
  df$rank <- rank(df$sd.id)
  ds1 <- merge(x = ds1, y = df, by = "id")#, suffixes = c("",""))
  ds1$y <- ds1$y - ds1$mean
  ds1$y <- (ds1$y - mean(ds1$y))/sd(ds1$y)
  ds1
}
```

Plot code

This initial plot is the one which appears in the main text, with 30 individuals simulated with 20 observations for each individual.

```
plot_CVp <- function(CVp, nID = 30, nrep = 20, seed = 42, label, x=TRUE, y=TRUE){
ds1 <- simulate(CVp = CVp, nrep = nrep)
plot(ds1$y ~ ds1$rank, ylab = '', xlab = '', axes=F, ylim = c(-5.5,5.5), xlim = c(0,30))
axis(1, at=c(0,10,20,30), labels=x)
axis(2, at=c(-4,-2,0,2,4), labels=y)
```

```
mtext(label, side=3, line = -1.5, at=1, adj=0, cex = 1.2)
box(bty='l')
}

# commented out commands to export plot
# tiff("Figure 2 - CVp Sim2.tiff", height = 4, width = 6, units = "in", res = 800)
par(mfrow=c(2,2), mar=c(0, 0, 1, 0.5) + 0.2, oma=c(3,3,0,0)+0.2)
plot_CVp(CVp = 0    , label = expression('a) CV'[P]*' = 0'), x=F)
plot_CVp(CVp = 0.25, label = expression('b) CV'[P]*' = 0.25'), x=F, y = F)
plot_CVp(CVp = 0.5  , label = expression('c) CV'[P]*' = 0.5'))
plot_CVp(CVp = 0.75, label = expression('d) CV'[P]*' = 0.75'), y=F)
mtext("Residual Deviance", side=2, line=2, cex = 1.2, outer=T)
mtext("Ranked rIIV", side=1, line=2, cex = 1.2, outer=T)
```

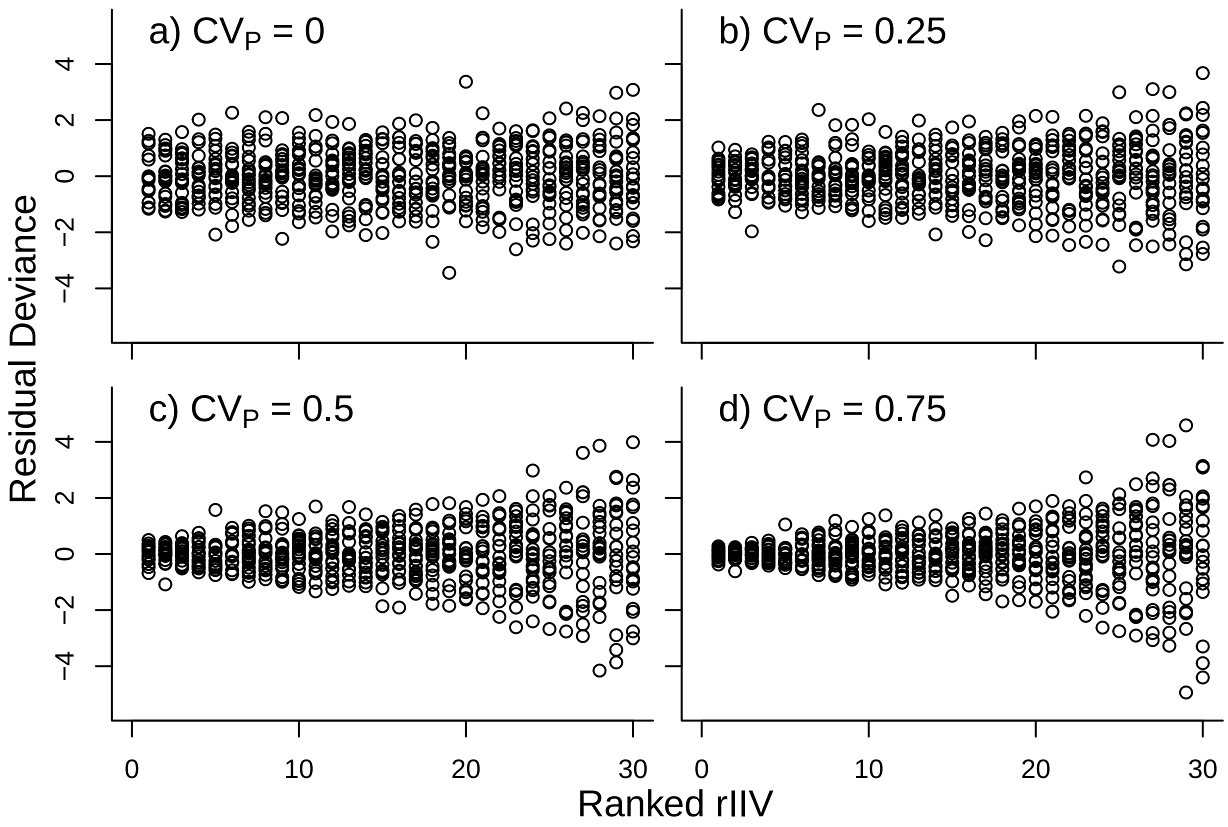

Small sample size

Finally, as stated in the main text, the illusion of among-individual variation can be seen when sample sizes are small. While the models do a good job parsing this sampling noise out (if distributional assumptions are met), this does mean that we should be careful in how we may perceive effects when exploring and visualising data. Here is the equivalent plot to above, with the exception that I have instead taken to lower threshold of datasets we considered – 5 observations per individual. Sampling more individuals will of course help the model's power, but will not help in this visualising exercise.

```
par(mfrow=c(2,2), mar=c(0, 0, 1, 0.5) + 0.2, oma=c(3,3,0,0)+0.2)
plot_CVp(CVp = 0    , label = expression('a) CV'[P]*' = 0'), x=F, nrep = 5)
```

```
plot_CVp(CVp = 0.25, label = expression('b) CV'[P]*' = 0.25'), x=F, y = F, nrep = 5)
plot_CVp(CVp = 0.5 , label = expression('c) CV'[P]*' = 0.5'), nrep = 5)
plot_CVp(CVp = 0.75, label = expression('d) CV'[P]*' = 0.75'), y=F, nrep = 5)
mtext("Residual Deviance", side=2, line=2, cex = 1.2, outer=T)
mtext("Ranked rIIV", side=1, line=2, cex = 1.2, outer=T)
```

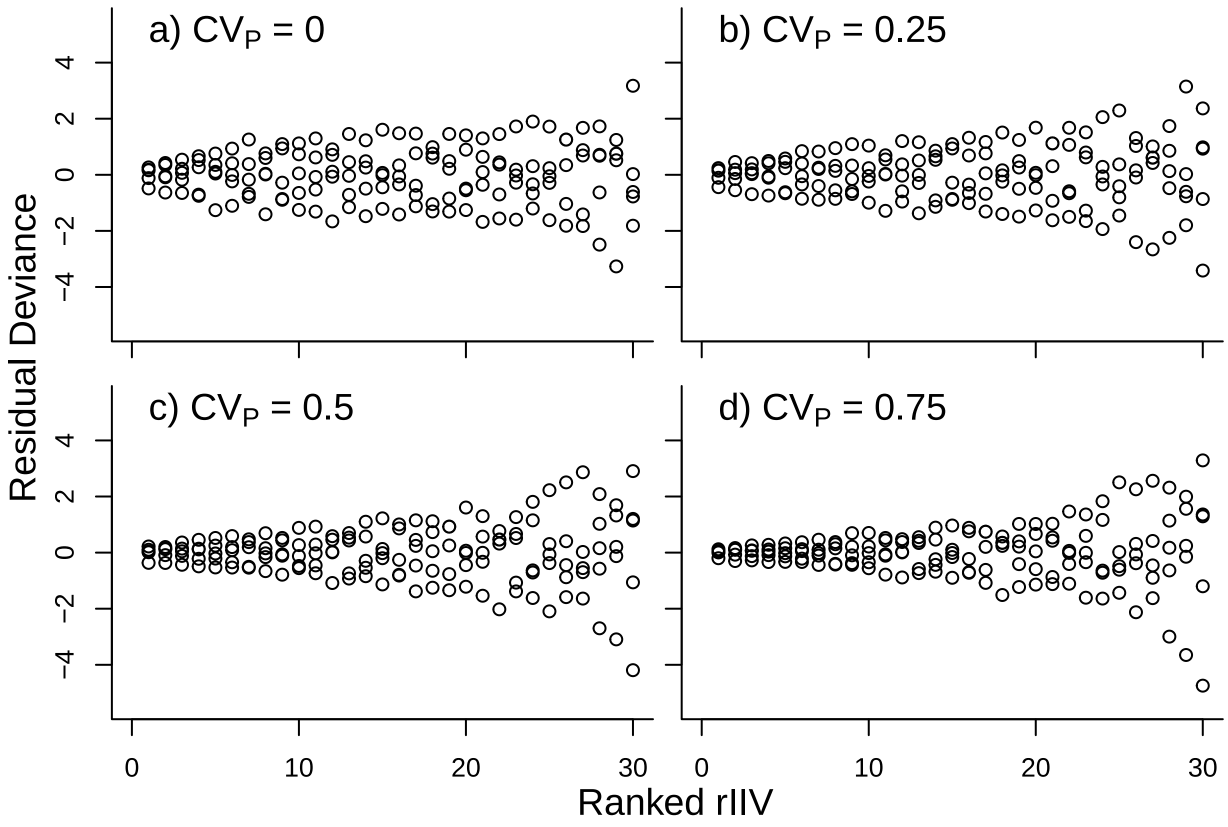

Supplement: Supplementary file 1 — Appendix S1 [file ECE3-11-7201-s001.pdf]
